# Should salvage surgery be considered for local recurrence after definitive chemoradiation in locally advanced non-small cell lung cancer?

**DOI:** 10.1186/s13019-016-0396-0

**Published:** 2016-01-19

**Authors:** Waldemar Schreiner, Wojciech Dudek, Sebastian Lettmaier, Rainer Fietkau, Horia Sirbu

**Affiliations:** Department of Thoracic Surgery, University Hospital, Friedrich-Alexander University Erlangen-Nuremberg, Erlangen, Germany; Department of Radiation Oncology, University Hospital, Friedrich-Alexander University Erlangen-Nuremberg, Erlangen, Germany

## Abstract

**Background:**

Incidence of local relapse after definitive chemoradiation (>59 Gy) for locally advanced non-small-cell lung cancer (NSCLC) is high, irrespective of high dose radiation applied. Experience with salvage lung resections in patients with locally relapsed NSCLC after definitive chemoradiation is limited. We present our series of salvage lung resections for local NSCLC relapse after curative–intent chemoradiation for locally advanced tumor.

**Methods:**

Nine consecutive patients with local tumor recurrence or persistence following definitive chemoradiation were reviewed. Kaplan-Meier analysis was used to assess patient survival.

**Results:**

All patients received definitive radiation (median dose 66.2 Gy) with concurrent chemotherapy. Tumor stage prior to chemoradiation was IIIA in 8 patients and IV in 1. In 4 patients tumor invaded the chest wall, in 2 the spine and in 1 the aorta. Median interval between chemoradiation and salvage resection was 30.2 weeks. Nine patients underwent 9 resections (6 lobectomies, 1 bilobectomy, 1 pneumonectomy and 1 bi-segmentectomy). One death occurred on the 12th postoperative day. Median overall survival was 23 months; postoperative 3-year survival was 47 %. Median progression-free survival was 21 months.

**Conclusion:**

Salvage lung resection for locally recurrent or persisted NSCLC in selected patients with locally advanced NSCLC following definitive chemoradiation is a worthwhile treatment option.

## Background

Since the mid-1990s the definitive chemoradiation therapy (CRT) has been a commonplace treatment for unresectable locally advanced NSCLC, or for resectable tumors in surgical high-risk patients [1, 2]. A local tumor relapse rate of up to 35 % can be expected in patients after definitive CRT and remains the dominant cause of death after the initial therapy [3]. There is no consensus on the effective local treatment strategy. Treatment options such as reirradiation, chemotherapy, cryo- and radiofrequency ablation, observation only and/or salvage surgery are applied [4–6]. The term “salvage surgery” is traditionally used in the multimodal management of the rectal and anal cancer as a part of “watch and wait” policy and is usually indicated for late local recurrence and/or for incomplete clinical response after neo-adjuvant chemoradiation [7–9]. Recently this term was adopted into the thoracic oncology and represents a considerable treatment option for local NSCLC recurrence after stereotactic body radiation in patients with early stage tumor [9–12]. Moreover, the salvage lung resection seems to be technically feasible in patients previously chemoradiated for locally advanced NSCLC [13–16]. Due to the limited experience, the patient selection criteria for salvage resections remain unclear. We report on our series of patients who underwent salvage lung resections for local NSCLC relapse or tumor persistence following the definitive CRT.

## Methods

The medical records of 9 consecutive patients, who underwent salvage lung resections at single institution between March 2011 and November 2013, were reviewed. All patients were treated for locally advanced NSCLC with a high dose radiation (>59 Gy) and concurrent platinum-based chemotherapy with curative intent. Operative selection criteria were as follow: local recurrence of the tumor after completion of definitive CRT, presence of the residual tumor after definitive CRT and cardiorespiratory fitness. The local recurrence (5 patients) was a new pulmonary lesion with high standardized uptake value (SUV). The residual tumor (4 patients) was defined as persistence of the tumor in the chest CT with persistently high SUV after complete definitive CRT. The preoperative diagnostics included total body computed tomography (CT) and fluorodeoxyglucose positron emission tomography (FDG PET), cranial MRI and cardiorespiratory function testing. Patient demographics, NSCLC stage at the time of diagnosis, pathologic characteristics of the resected tumor, length of hospital stay, perioperative complications and mortality were reviewed. The overall survival was calculated from the time of lung cancer diagnosis. The disease free-survival was an interval between completion of CRT and detection of the tumor relapse. The progression-free survival was defined as the interval between the salvage resection and locoregional or distant recurrence of the tumor. The long-term survival was a 3-year survival after lung resection.

The statistical data analysis was performed using SPSS (version 21.0 for Windows; IBM SPSS, Inc., Chicago, IL). Descriptive statistics were applied for patient characteristics, surgical and oncologic outcome. Survival rates were calculated using the Kaplan-Meier method and compared with a long-rank test. Differences were considered to be statistically significant for p values of <0.05.

## Results

The median age at the time of salvage resection was 56.2 years; 8 patients (89 %) were younger than 65. Six patients (67 %) were male. Detailed pre-CRT patient characteristics are shown in the Table 1. The median radiation dose applied to the primary tumor was 66.2 Gy (range 59.4 – 72 Gy). All patients received concurrent platinum-based chemotherapy in combination with vinorelbine (in 5 patients) or etoposide (in 4 patients).

**Table 1:** Pre-treatment characteristics

Patients	Age (Y)/Gender	ECOG	Tumor Size (mm)	cTNM-Stage	Histology	Radiotherapy	Chemotherapy	Reason for def. CRT
1	52/F	0	90	cT4N1M0	Adeno	59.4	Cisplatin/Vorelbine	
2	64/M	1	75	cT3N1M0	Squamous	66.6	Cisplatin/Vinorelbine	limited lung function
3	63/M	1	49	cT4N1M0	Adeno-squam	72	Carboplatin/Vinorelbine	Liver transplantation
4	62/M	2	48	cT3N1M0	Adeno-squam	66	Cisplatin/Etoposide	Liver transplantation
5	55/F	0	56	cT4N1M0	Adeno	59.4	Cisplatin/Etoposide	
6	37/M	0	37	cT4N0M1	Adeno-squam	66	Carboplatin/Vinorelbine	Oligometastasis in iliacal bone
7	71/M	1	56	cT2N2M0	Adeno	72	Cisplatin/Etoposide	limited lung function
8	55/F	0	82	cT4N0M0	Adeno	66.2	Cisplatin/Vinorelbine	
9	53/M	0	87	cT4N0M0	Adeno	70.2	Cisplatin/Vinorelbine	

**Fig. 1:**
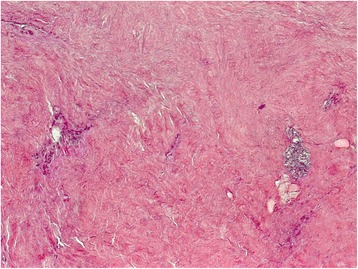
Histopathologic finding with excessive fibrosis without the evidence of active tumour cells cuased by definitive chemoradiation

Detailed post-treatment characteristics: interval between CRT and salvage resection, type of resection, tumor stage and size, percentage of viable cancer cells and postoperative complications are shown in the Table 2. The median interval between completion of the CRT and salvage surgery was 30.2 weeks (range 12.4 - 165.7 weeks). Eight lobar or greater resections (5 lobectomies, 1 lobectomy combined with segmentectomy, 1 bilobectomy, 1 pneumonectomy) and 1 bi-segmentectomy (in patient with reduced lung function) were performed. All lung resections were completed by a systematic hilar and mediastinal lymph node dissection. Seven patients (78 %) required extended resections (1 chest wall resection, 2 vertebral column resection and reconstruction, 1 replacement of descending aorta). The complete tumor resection (R0) was achieved in all 9 cases and the pathologic examination demonstrated viable tumor cells in 8 specimens (88 %). In one patient 27 months after definitive CRT a SUV of 3.3 was detected. But only inactive tumor tissue with extensive necrosis surrounded by fibrosis was found in the histo-pathologic examination [Fig. 1].

**Table 2:** Post-treatment characteristics

Patients	Interval between def. RCT and surgery (month)	Surgical procedure	ypTNM-stage	Tumor size (mm)	Viable tumor cells (%)	Perioperative complication
1	27.4	upper bilobectomy with chest wall and Th1-2 resection	T0N0M0	30	0	
2	3	extended lobectomy with chest wall	T3N0M0	55	60	post-resectional empyema, ARDS
3	10.8	pneumectomy with aortic arch	T4N0M0	70	70	multi-organ failure
4	38.7	S1/2- bisegmentectomy	T2aN0M0	50	60	
5	7.8	lobectomy with chest wall	T3N0M0	50	10	
6	6.7	lobectomy with chest wall and T1-3 resection	T4N0M1	10	50	
7	6.7	lobectomy	T0N2M0	50	10	
8	3.5	upper bilobectomy with chest wall	T2bN0M0	54	5	
9	5.7	lobectomy with chest wall	T2bN0M0	62	30

The median duration of the surgery was 3 h 54 min (range 2 h 02 min - 8 h 26 min). The median blood loss was 400 ml [Range, 250–550 ml]. One patient developed an adult respiratory distress syndrome (ARDS) postoperatively and required prolonged mechanical ventilation. He was also re-operated for a post-operative empyema. One patient died on the 12th day following lobectomy with partial aortic arch resection, due to multiple organ failure.

The median postoperative length of hospital stay was 20 days (range 9 – 68 days). The median postoperative follow up was 30 months (range 5.7 – 41.2 months). In two patients a locoregional recurrence 10.3 and 41 months postoperatively was evident. In other four cases distant metastases occurred. Femoral bone and adrenal gland metastases were diagnosed in one patient each 6.2 and 18.3 months, respectively. Two patients developed multiple brain metastases 2.5 and 21.9 months postoperatively. During the follow-up interval 3 patients died due to tumor progression 12, 14 and 22.9 months after the salvage resection, whereas the patient with adrenal gland metastasis remained alive and required stereotactic body radiotherapy.

**Fig. 2:**
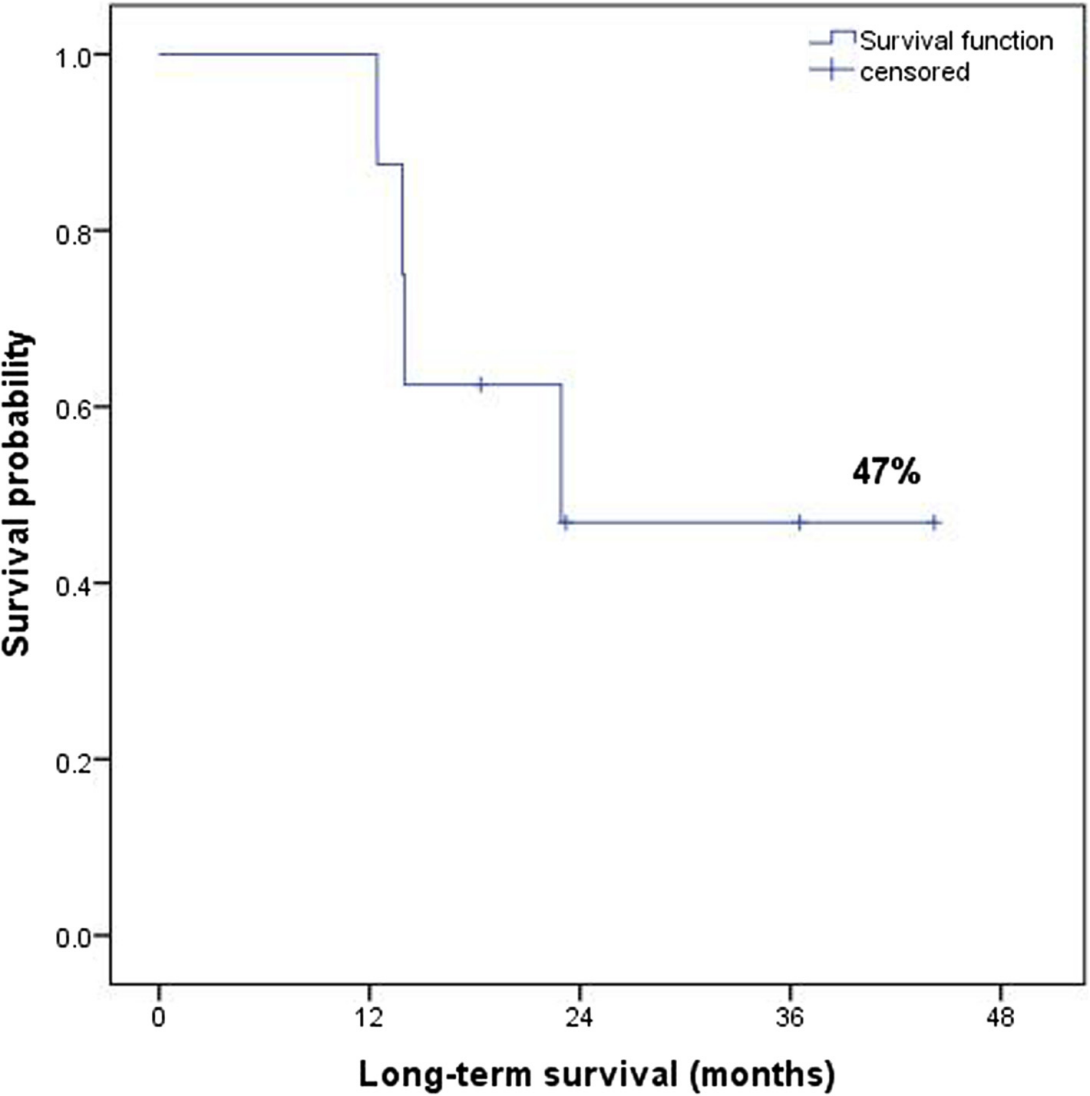
Overall long-term and progressive-free survival after salvage surgery following definitive chemoradiation

The median overall survival was 63.9 months (range 21.5 – 63.9); the median post-chemoradiation survival was 61.6 months (range 15.3 - 61.6 months). Detailed outcome is demonstrated in the Table 3. The median postoperative long-term survival was 23 months (range 10.8 - 38 months) and the 3 years-survival rate was 47 % [Fig. 2]. The median progression-free survival was 21 months (range 2.5 - 38 months) and 3-year progressive-free survival rate was 40 %.

**Table 3:** Postoperative outcome

Patients	Follow-up after surgery (months)	Disease free after surgery	Local recurrence	Distant recurrence	Tumor relapse	Outcome status
1	36.5	36.5				alive tumor free
2	10.2	6.2		femur bone		died tumor related
3						died, postoperatively
4	22.9	21.9		diffuse distant metastasis		died tumor related
5	18.3	18.3		adrenal gland metastasis		alive
6	13	10.3	chest wall ipsilateral			dead tumor related
7	44.2	41	lymph node metastasis ipsilateral			alive
8	18					alive tumor free
9	14	2.5		multiple brain metastasis		dead tumor related

## Discussion

The rates of local NSCLC recurrence after definitive CRT are even as high as 85 % at 1 year [17, 18]. Pain, dyspnoea, haemoptysis and cough are frequent manifestations [20]. The local NSCLC relapse remains the leading death cause after the initial therapy [3].

The difficulties in distinguishing between tumor recurrence and fibrosis or inflammatory changes on the computed tomography (CT) and FDG-PET are reported [24, 25, 26]. Dense consolidations are typical for radiation pneumonitis or fibrosis. However, the tumor re-growth must also be suspected and considered differential diagnosis [2]. The increased SUVmax values should require further clearing by CT-guided or trans-bronchial biopsy. On the other side, the excessive fibrosis or tumor necrosis has to be expected. The fine-needle aspiration biopsy provides limited pathologic information. Therefore, distinguishing between radiation- and tumor-induced fibrosis may lead to false-negative findings. In some patients without tumor recurrence, a moderate FDG hypermetabolic activity may persist up to 2 years after CRT [22]. Therefore, the optimal treatment strategy, when the histology is unclear, remains undefined and suspected recurrence requires at least a comprehensive evaluation [21]. The right time window for the tumor biopsy and for beginning the therapy is crucial. If only focal tumor nest surrounded by necrotic mass is evident, the further course of the “remaining tumor” is unpredictable. Presence of the tumor cell nest must not indicate the recurrence as those cells may become necrotic [19, 20]. On the other side, awaiting the radiological obvious relapse significantly decreases the overall and the progressive-free survival time [14, 15]. Particularly, in patients with extended relapse invading the neighbouring structures, the delay in decision making decreases the chance of the tumor resectability. The biological nature of the local NSCLC relapse is less malignant in comparison to the distant recurrence. Therefore the effective local treatment is likely to be associated with prolonged overall survival and long-term disease control. In addition, some authors recommend the salvage resections immediately after detecting the local recurrence [2].

The choice of a re-treatment strategy for local recurrence after the definitive CRT remains challenging. Only few therapeutic alternatives have been reported. A systematic literature review regarding the second line chemotherapy efficacy for relapsed NSCLC shows a moderate symptom control and response rates for several anticancer drugs. The median long-term survival and progressive-free survival remain under 9 and 6 months respectively [21]. In addition, Hanna et al. report only a minimal increase in survival following re-chemotherapy [22]. The reported median long-term survival after reirradiation ranges from 5 to 14 months, whereas the 2-year survival rates and the 2-year progression-free survival were 27 % and 21 % respectively [17, 20]. Radiation tolerance of the lung, esophagus and the spinal cord limit the radiation dose that could be applied to the recurrent tumor. Particularly, the myelopathy and the late lung fibrosis have to be avoided. Jeremic et al. reviewed 11 studies of conventionally fractionated external beam reirradiation for recurrent NSCLC. The analysis showed improved overall survival after higher doses compared with low-dose reirradiation. However, this was associated with an increased rate of pneumonitis and esophagitis [3]. Toxicity strongly results from applied total and cumulative median radiation dose. However, locoregional and distant tumor control remains disappointing [22]. A radiofrequency ablation and cryoablation of the local recurrence is limited by the position of the lesion and the tumor size. The percutaneous cryoablation is recommended for more central lesions, in proximity to the large vessels [5]. The radiofrequency ablation is usually performed for lesion smaller than 3 cm. The long-term local control is dependent on the tumor size and remains, particularly in cases with lesion greater than 3 cm, unfavourable. Due to quick tumor progression frequently repeated invasive procedures are required [6].

Recently published reports tend to recommend salvage lung resections as feasible treatment option in absence of other management strategies for recurrent NSCLC. Due to limited experience, the patient selection criteria for “post-radiotherapy” salvage lung surgery are not clearly defined. Low postoperative mortality and complication rates despite high-dose stereotactic body radiation support the idea of salvage surgery for local recurrence in early stage NSCLC [10, 11]. Four other reports suggest that salvage surgery is associated with prolonged survival in patients with locally recurrent or persistent tumor after definitive CRT in locally advanced NSCLC [13–16]. Bauman et al. state that the risk of salvage resection is proportional to the intensity of fibrotic response after the high-dose radiation and to the interval between radiation and resection. However, even in high-risk patients the salvage surgery was technically feasible, with reasonable results, also when performed after long-time interval [14]. In addition, some authors identified salvage lung surgery as the best option for patients with local tumor relapse, resulting in a prolonged survival [13, 23].

Kuzmik et al. reviewed 14 patients who completed definitive chemoradiation with median dose of 57 Gy. After median interval between chemoradiation and surgery of 33 months [range 0–169 months] local recurrence was identified in 54 % of the cases, locoregional in 15 % and distant in 31 %. Viable tumor was found in all cases (100 %). The median postoperative survival was 9 month with the 2-year survival rate of 49 % [13].

Bauman et al. described 24 patients treated initially with definitive chemoradiation with a median radiation dose of 63.9 Gy. The median time between radiation and surgery was 21 weeks. Nineteen patients (78 %) had pathological proof of viable tumor cells in the resection specimen. The median survival time for entire cohort was 30 months with estimated 3-year survival of 47 % [14].

Yang et al. described a cohort of 31 patients with various indications for salvage resections. The chemoradiation was performed in 90 % of the cases, with median radiation dose of 60Gy. The median interval between radiation and surgery was 17.7 weeks. Viable tumor cells were identified in 19 (61 %) specimens. The median survival time for entire group was 32.5 months with 3- and 5-year survival rates of 42 % and 31 %, respectively. The patients with residual disease expected a median survival time of 20 months. Median survival time in patients with complete pathologic response was 60 months and differed statistically significant (*p* = 0.03) [15].

Casirhagi et al. described an extended salvage resection in a group of 24 patients. The median time between the definitive chemoradiation (mean 51 Gy) was 12 weeks and was therefore close to the time interval for surgical resection after the induction therapy. Viable tumor was found in 90 % of the cases. The postoperative 3-years survival was 42 % [16].

Detailed study characteristics are summarized in the Table 4.

**Table 4:** Overview to available results for salvage lung surgery after definitive chemoradiation in locally advanced NSCLC

Autor	Year	Number	Median Age (Y)	Median Radiation dose (Gy)	Median follow up (month)	Interval between RCT und salvage surgery (month)	Interval between RCT und salvage surgery (weeks)	Extended resections (%)	Median OS (month)	3Y-Survival (%)	5Y-Survival (%)	Median PFS (month)
Bauman	2008	24	60	63.9	29	4.6	20.6	21	30	47		12
Kuzmik	2013	14				33		21	9	49 (2Y)		
Casiraghi	2014	43	64	57	21	2.7	12.2	56		42		
Yang	2015	31	58	60	40	4	18	16	32.5	42	31	10
Our study	2015	9	56	66	30	6.7	30.2	78	23	47		21

Our group included only those patients with uncontrolled local disease (local relapse or residual tumor following definitive CRT). The median disease-free survival in our series was similar to the Bauman’s group (6.7 vs. 5 months). In contrast, Kuzmik et al. performed salvage resections in patients with tumor recurrence in the contralateral lung (31 %) and in the other ipsilateral lobe (15 %). Only 54 % out of 14 salvage lung resections were performed for local recurrence. The median disease-free survival for all patients was 33 months [13]. The ratio of extended salvage resections involving the neighbouring organs was in our group higher (21 % by Bauman vs. 21 % by Kuzmik vs. 56 % Yang and 78 % our group). Remarkable, despite the described differences the median long-term survival and estimated 3-year survival rates in our group were comparable with other series.

Majority of our patients developed locoregional (25 %) or distant recurrence (50 %) during the follow-up interval. Therefore the long-term disease control remains crucial and interdisciplinary decision making regarding further treatment is essential. Some important points of agreement are as follow: 1) the salvage lung surgery should be performed in patients with no other treatment alternatives; 2) the experience in this new field in the thoracic surgery remains limited; 3) a careful patient selection, particularly for extended resections, is fundamental.

Our study has a number of limitations. Small number of salvage resections was performed and the data was collected retrospectively. A control group of patients with local NSCLC recurrence after definitive CRT managed without salvage surgery was not available for the comparison.

Salvage extended thoracic surgery may represent a good therapeutic alternative in well selected cases with adequate pulmonary reserve and good performance status. The individual patient targeted approach is essential and all alternative treatment options should be discussed interdisciplinary.

## Conclusions

To date, the role of the postradiochemotherapy surgical lung salvage in patients with local relapse after definitive chemoradiation for locally advanced NSCLC is poorly defined. However, salvage resections are technically feasible and are associated with reasonable survival in selected cases. The appropriate long-term tumor control determines the patient outcome. Therefore, an interdisciplinary consensus and adequate patient selection criteria for salvage surgery are required.
